# Eco-Sustainable Silk Fibroin/Pomegranate Peel Extract Film as an Innovative Green Material for Skin Repair

**DOI:** 10.3390/ijms23126805

**Published:** 2022-06-18

**Authors:** Marianna Barbalinardo, Marta Giannelli, Ludovica Forcini, Barbara Luppi, Anna Donnadio, Maria Luisa Navacchia, Giampiero Ruani, Giovanna Sotgiu, Annalisa Aluigi, Roberto Zamboni, Tamara Posati

**Affiliations:** 1 National Research Council, Institute for the Study of Nanostructured Materials (CNR-ISMN), Via P. Gobetti 101, 40129 Bologna, Italy; marianna.barbalinardo@ismn.cnr.it (M.B.); giampiero.ruani@cnr.it (G.R.); 2 National Research Council, Institute for Organic Synthesis and Photoreactivity (CNR-ISOF), Via P. Gobetti 101, 40129 Bologna, Italy; marta.giannelli@isof.cnr.it (M.G.); ludovica.forcini@studio.unibo.it (L.F.); marialuisa.navacchia@isof.cnr.it (M.L.N.); giovanna.sotgiu@isof.cnr.it (G.S.); 3 Department of Pharmacy and Biotechnology, University of Bologna, Via San Donato 19/2, 40127 Bologna, Italy; barbara.luppi@unibo.it; 4 Department of Pharmaceutical Sciences, University of Perugia, Via del Liceo, 1, 06123 Perugia, Italy; anna.donnadio@unipg.it; 5 Department of Biomolecular Sciences, School of Pharmacy, University of Urbino, Piazza del Rinascimento 06, 61029 Urbino (PU), Italy; annalisa.aluigi@uniurb.it

**Keywords:** silk fibroin, pomegranate waste, bioactive films, skin repair, circular economy

## Abstract

Skin disorders are widespread around the world, affecting people of all ages, and oxidative stress represents one of the main causes of alteration in the normal physiological parameters of skin cells. In this work, we combined a natural protein, fibroin, with antioxidant compounds extracted in water from pomegranate waste. We demonstrate the effective and facile fabrication of bioactive and eco-sustainable films of potential interest for skin repair. The blended films are visually transparent (around 90%); flexible; stable in physiological conditions and in the presence of trypsin for 12 days; able to release the bioactive compounds in a controlled manner; based on Fickian diffusion; and biocompatible towards the main skin cells, keratinocytes and fibroblasts. Furthermore, reactive oxygen species (ROS) production tests demonstrated the high capacity of our films to reduce the oxidative stress induced in cells, which is responsible for various skin diseases.

## 1. Introduction

Skin diseases such as atopic dermatitis, eczema and psoriasis; and skin injuries such as chronic wounds (e.g., diabetic foot and bed sores), represent an increasing problem all over the word: they collectively have an estimated 1-year prevalence of up to 20% among children and 2–10% among adults [1,2]. Beyond genetic predisposition, several factors contribute to the development of dermatitis and chronic wounds, such as diet, infections, inflammatory processes, alcohol abuse, smoking, ionizing and solar radiation (UVA and UVB rays), air pollution, drugs and toxic substances in general [3]. Such stimuli induce oxidative stress, which is excessive production of (ROS) that trigger pathological mechanisms altering the normal physiological parameters of cells. ROS are produced in the body’s cells during metabolic processes, and in physiological conditions, they are necessary for the correct functioning of cells, helping the immune system with inflammation, wound healing and defense against germs. In chronic dermatoses, the ROS production exceeds the capacity of antioxidant defense in the skin (by enzymes such as superoxide dismutase and catalase); thus, their modulation is important in maintaining skin homeostasis [4,5,6]. In the last few decades, various types of antioxidants (more or less effective) have been introduced for the prevention and treatment of chronic wounds and dermatitis, such as vitamins A, C and E; polyphenols; panthenol; lipoic acid; coenzyme Q10; lycopene; and several extracts—for example, from *Ocimum basilicum*, *Trifolium pretense* and *Stellaria media* [7,8]. Recently, researchers have especially focused on finding new therapeutic strategies in the field of natural products [9,10,11]. In this respect, the use of waste materials is a crucial point—especially from the perspective of a circular economy, for which it is necessity to increase resource efficiency. Using agricultural and industrial by-products as raw materials is essential for maintaining human and environmental well-being [12]. Pomegranate fruit gained considerable recognition as functional food in the modern area. Indeed, much literature highlights the potential health benefits of pomegranate juice and fruit extracts [13]. Particular attention is paid to pomegranate by-products in terms of peel, seed and pomace derived from the pomegranate juice and jam industry. This is because it has been demonstrated that pomegranate wastes have, like the juice, marked antioxidant, antimicrobial, anti-mutagenic and anti-inflammatory properties due to the high presence of polyphenols such as tannins and flavonoids [14]. For example, recently, it has been reported that pomegranate extracts showed antiproliferative activity against human bladder cancer cells (T24) [15] and against neuroblastoma and breast cancer cells [16]. Pomegranate by-products and derived extracts have been widely used for food applications in order to improve nutritional quality or prolong shelf life [17]. Nevertheless, literature dealing with specific repurposing of pomegranate peel powder as an antioxidant in skin repair is still not very abundant. Therefore, effective and exciting research progress is of the utmost importance to addressing these challenges in the future. For skin repair, a matrix capable of both releasing the active ingredient in a controlled manner and mimicking the structure and biological functions of native extracellular matrix (ECM) is desirable [18]. ECM is mainly composed of proteins and polysaccharides and it is the bulkiest component of the dermal layer. It plays a key role in the process of skin repair, especially in chronic wounds. Several natural proteins, such as collagens, elastin, keratins, albumin and fibrin, have been used as tissue scaffolds [19,20,21]. Among these, silk fibroin (SF), extracted from *Bombyx mori* cocoons, is reported to be a high performant substrate for cell adhesion and proliferation due to its well-known advantageous properties, such as high tensile strength, controlled biodegradability, controlled drug release, biocompatibility and non-inflammatory characteristics [22,23,24,25]. Regenerated silk films are already used effectively to promote angiogenesis, thereby helping in the wound healing process by facilitating re-epithelialization and collagenization [26,27]. Several natural antioxidants, such as quercetin, grape seed extract, vitamin C, tannins, oleuropein and rutin, have been incorporated or absorbed into SF in order to obtain bio-based materials for biomedical applications [28,29,30]. Recently, we developed an active pad based on pomegranate peel powder and reduced the amount of SF, used as glue, for food packaging applications [31]. However, the use of SF in skin repair with a bioactive antioxidant product derived from waste material such as pomegranate peel had before now not been studied. Hence, in the present study, we combined for the first time SF and pomegranate peel extracts (EPP)—aqueous solutions—to obtain bioactive free-standing films. The resulting films were fully characterized and tested in vitro with the main skin cells, keratinocytes and fibroblasts. They demonstrated their eligibility as innovative, ecofriendly, eco-sustainable and bioactive materials for chronic wound and dermatitis treatments.

## 2. Results and Discussion

### 2.1. Characterization of Pomegranate Peel Extracts (EPP)

**Figure 1:**
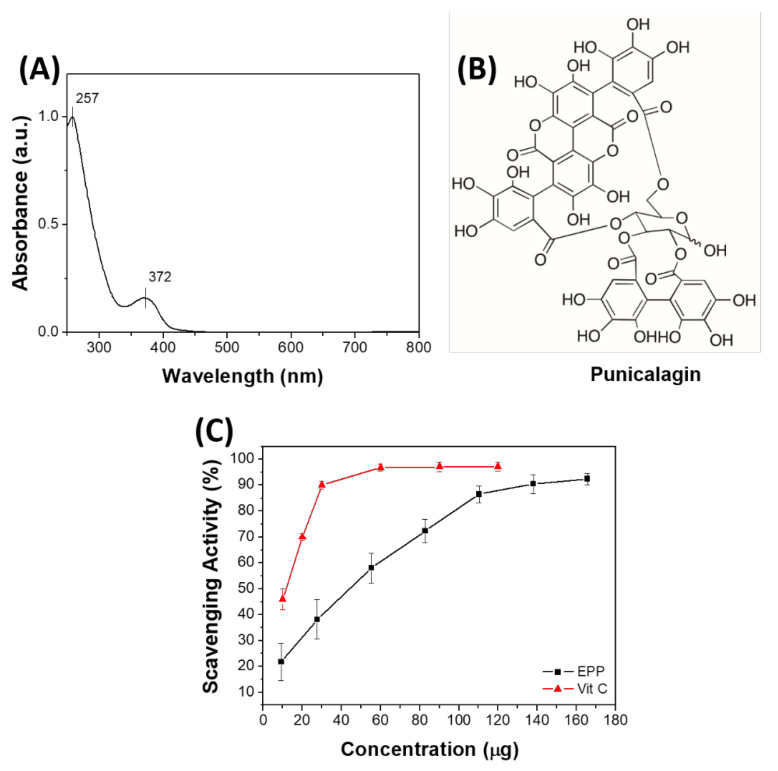
(**A**) UV–Vis spectrum of EPP; (**B**) molecular structure of punicalagin; (**C**) radical scavenging activity of EPP compared to that of vitamin C.

The antioxidant compounds present in the pomegranate peel were extracted by using water as solvent and following the procedure described in the Section 3. The extracted solution of EPP was first analyzed by UV–Vis spectroscopy. The UV–Vis spectrum of EPP (Figure 1A) shows two bands: a more intense one at 257 nm and a less intense one at 372 nm. When comparing the EPP spectrum with that of punicalagin (Figure 1B), the main component of pomegranate [32] (λ-max at 258 and 380 nm), we observed similar absorbance [33,34]. To get deeper insights on EPP, HPLC-DAD-MS analyses were performed. A 3.75 mg/mL aqueous solution sample was prepared and analyzed by HPLC-DAD-MS. The HPLC chromatograms recorded at λ 260 and 380 nm are reported in Figure S1A. HPLC profiles at 260 and 380 nm showed two major peaks at RT = 9.74 and 11.24 min in a ca. 1:2.5 ratio, with maxima at λ 255 and 375 nm and 255 and 380 nm, respectively (Figure S1B). HPLC-MS analysis showed the same molecular ion [M-1] *m*/*z* 1083 and similar mass profile for both peaks (Figure S1C). On the basis of the acquired data and in agreement with previous reports [34], the two peaks can be attributed to punicalagin α and β anomer, respectively. HPLC-DAD-MS analyses allowed also identifying most of the minor chromatographic peaks. By comparing the spectroscopic and spectrometric data acquired, and the relative retention times, with previously reported data, [34,35] nine more tannins present in EPP were identified (details in Figure S3 and Table S1). Thus, the radical scavenging ability of the EPP was evaluated in vitro based on the DPPH assay. Figure 1C shows the scavenging activity of EPP (black line) compared to that of a well-known antioxidant agent, vitamin C (red line). Interestingly, the antioxidant activity of EPP is of the same order of magnitude of that of vitamin C (EC50 EPP—40 μg and EC50 vitamin C—13 μg). Of note is that the DPPH test confirmed the complete extraction of antioxidants from pomegranate powder; indeed, the extraction residue did not show any antioxidant activity, even at high concentrations (~150 mg in 17 mL of DPPH) (Figure S3). After 20 min of incubation, the absorbance of DPPH in the presence of the residue (A = 1.3718) was similar to that of only DPPH (A = 1.3867).

### 2.2. Preparation of SF-EPP Films

**Figure 2:**
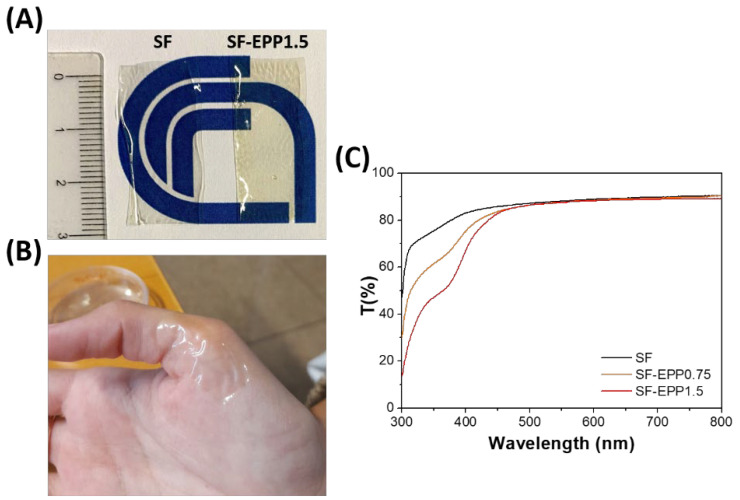
(**A**) Picture of pristine SF and SF-EPP films (1 mL deposited on 1 cm × 3 cm) with the highest EPP content (1.5% vs. SF); (**B**) SF-EPP1.5 adapted to the skin; (**C**) transmittance of SF (black line), SF-EPP0.75 (orange line) and SF-EPP1.5 films (red line).

Figure 2A shows the SF films with and without EPP. The thickness of the films was about 50–60 µm, and as reported in literature, [36] the addition of glycerol (20% wt/wt vs. SF) induced water insolubility of the protein, leading to an increase in the stability of the resulting SF-EPP film. As can be seen in Figure 2A,B the resulting films appeared optically transparent, macroscopically homogeneous and able to adapt to the folds of skin.

### 2.3. Optical and Structural Characterization of SF-EPP Films

**Figure 3:**
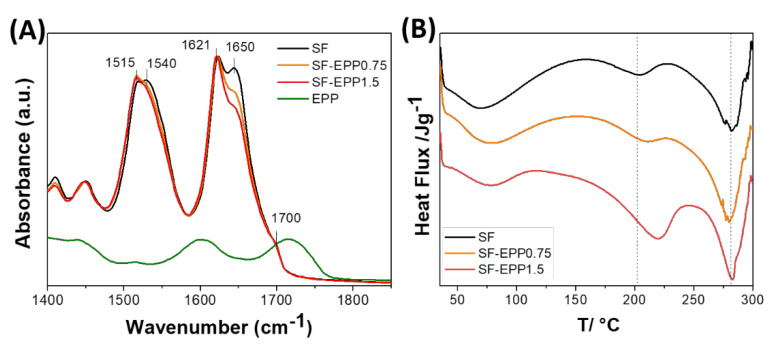
(**A**) FTIR spectra of pristine SF (black line), SF-EPP0.75 (orange line) and SFEPP1.5 (red line) films and EPP (olive line) in the 1400–1900 cm^−1^ range. (**B**) DSC curves of SF (black line), SF-EPP0.75 (orange line) and SF-EPP1.5 (red line) films.

In skin disease treatments, the product’s aesthetics (such as appearance, aroma and texture) can greatly contribute to patient compliance. An important property of SF-based films is their optical transparency in the visible range. [37,38] It makes them an excellent substrate for topical and transdermal formulations. In this respect, as well as visually, the transparency of SF-EPP films was investigated by light transmittance characterization. Figure 2C shows the transmission spectra of SF and SF-EPP films. Similarly to the SF film, SF-EPP films were highly transparent (86–90%) in the visible region (450–800 nm), and consistently less so below 450 nm as a result of antioxidant absorption, in agreement with the previous absorption spectra (Figure 1A). The ATR spectra of pristine SF and SF-EPP films are reported in Figure 3A. The IR absorption bands in the 1400–1800 cm^−1^ spectral region mainly arise from vibrational modes of the polypeptide amide group, and thus can be related to the secondary structure of the protein. Changes in the protein’s secondary structure, induced by the presence of the extract, can then be followed by monitoring the infrared absorption. The peptide group gives rise to characteristic bands: the amide I, between 1600 and 1700 cm^−1^, mainly due to the stretching of the C=O bond, and the amide II, between 1500 and 1600 cm^−1^, related to the bending vibration of the NH and to the stretching vibration of the CN bond [38,39]. The water-soluble Silk I amorphous conformation related to the α-helix and random coil structures has intense absorption bands around 1550 and 1650 cm^−1^, whereas the crystalline water-insoluble Silk II conformation, associated with the β-sheet structures, has absorption bands around 1515, 1620 and 1700 cm^−1^ [39]. Figure 3A shows that the ATR spectra of SF and SF-EPP films are characterized by the typical absorption bands due to the water-insoluble β-sheet structures induced by the presence of glycerol. Furthermore, in the SF-EPP samples, the bands at around 1540 and 1650 cm^−1^ decreased in intensity, probably due to the reduction in α-helices and random coil structures, and not the contribution of the EPP band at 1720 cm^−1^, which would have also affected the protein band at 1700 cm^−1^. To better quantify the changes observed in the ATR spectra, we performed an analysis of secondary structure elements using deconvolution of amide I and amide II bands. The results of curve fitting are shown in Figure S4 and Table S2. Measurements showed that the SF-EPP samples contained lower amounts of α-helical structures (~31% for SF-EPP0.75 and ~29% for SF-EPP1.5) compared with SF only (~60%).

In order to understand the influence of EPP on the crystallization of the SF, thermal behaviors of the SF and SF-EPP films were investigated using DSC. Figure 3B shows an endothermic peak ranging from 60 to 100 °C due to the water adsorption and a second endothermic peak at around 200 °C due to the transition of unstable noncrystalline structures to β-sheets [40]. This latter peak goes from about 200 °C for pure SF to ca. 220 °C for SF-EPP1.5; the enthalpy value is 13.78 for SF and 74.78 J g^−1^ for SF-EPP1.5. The larger enthalpy reflected the higher degree of crystallization, meaning that the SF-EPP film was more crystalline than pure SF, confirming the ATR hypothesis on the reduction of the amorphous secondary structures. The polymer molecular chains of the SF-EPP were more packed due to the interactions between EPP and SF, which were crucial for crystallizing SF and increasing its melting temperature. Above this crystallization temperature, all films started to degrade, despite an unchanged endothermic peak at around 257 °C.

### 2.4. Mechanical Properties of SF-EPP Films

The mechanical properties, in particular, the Young’s modulus (E), tensile strength (TS) and elongation at maximum strength (ε), of SF-EPP films are presented in Table 1. At ambient conditions, the Young’s modulus of SF-EPP films was slightly higher than that of pristine SF, likely due to the higher crystallinity of fibroin after incorporation of EPP, as seen from DSC data. TS and ε did not significantly change with the presence of EPP. The amount of EPP incorporated was not probably sufficient to induce an important structural change with consequent drastic changes in the mechanical parameters. Interestingly, when we increased relative humidity (RH) and temperature, mimicking some of the injured skin conditions (T = 37 °C and RH = 80%), the films showed lower values for mechanical parameters with respect to the values determined at low RH (33%), making them more flexible (see Table 2). These results may be caused by possible interactions between phenolic compounds (present in the EPP), SF and the H-bonding network due the water molecules adsorbed at high relative humidity, which are expected to increase film plasticity, since water also acts like a plasticizer and forms crosslinks that may lead to the formation of more cohesive and flexible matrices [41]. The polymeric matrix becomes less dense at 80% RH and under stress; movements of polymeric chains were facilitated. The mechanical behavior of the films at high RH depended on the concentration of EPP, and the specific interactions between components determined the effective attraction forces between polymeric chains. The addition of EPP produced a significant increase in the elastic modulus, resulting in stiffer films, but neither the tensile strength nor the elongation at maximum strength was negatively affected. SF-EPP maintained adequate mechanical strength and extensibility, conserving its integrity in a damaged-skin-like environment. 

### 2.5. Stability and Biodegradability of SF-EPP

**Figure 4:**
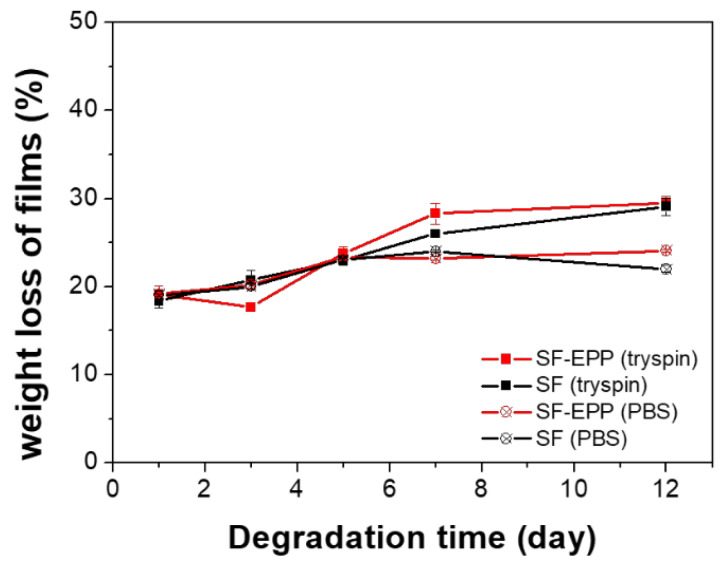
Enzymatic degradation profiles compared with PBS dissolution profiles of pure SF (black lines) and SF-EPP films (red lines).

For stability and release testes, we focused only on the SF-EPP1.5 sample, due to its similar properties to SF-EPP0.75. The stability and biodegradability of SF and SF-EPP1.5 films were tested in vitro by degradation with trypsin, while using PBS as a blank. Figure 4 shows that all samples immediately lost about 20% of their weight, which in PBS remained unchanged for up to 12 days. In the proteolytic solution it increased slightly, by up to about 30%, independently of the EPP’s presence. The constant initial weight loss in all samples could have been due to the rapid diffusion of glycerol (20% wt/wt vs. SF) into the buffer solution. Given the weight losses in PBS and with trypsin, the effect of the enzyme up to the 12th day is minimal, indicating the high stability and reduced biodegradability of the samples, in agreement with ATR data.

### 2.6. Release of Antioxidants from SF-EPP Film

**Figure 5:**
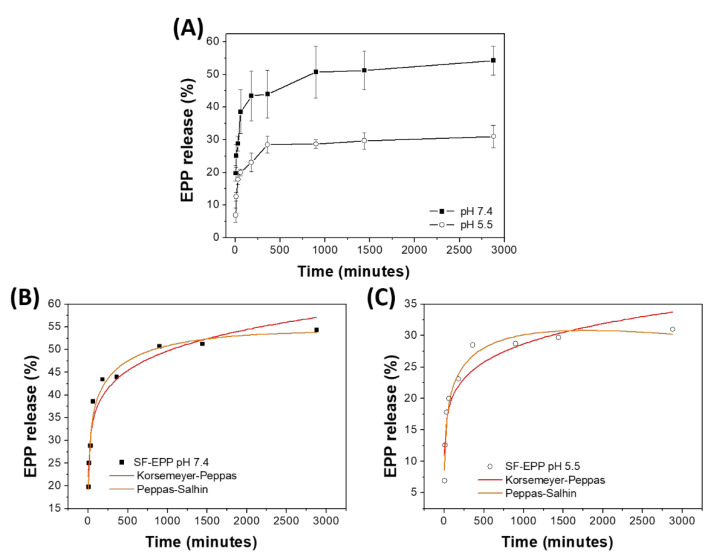
(**A**) EPP release profiles of SF-EPP1.5 at different pHs; (**B**,**C**) EPP release profiles fitted by Korsemeyer-Peppas and Peppas–Sahlin models for pH 7.4 and 5.5, respectively.

**Figure 6:**
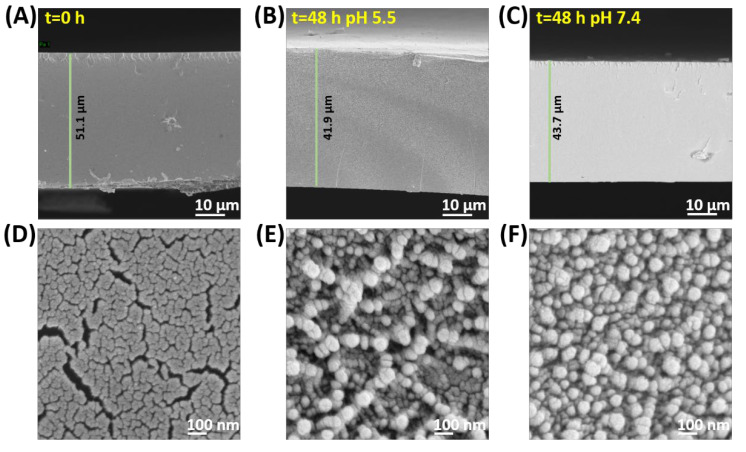
SEM images of pristine SF-EPP film (**A**,**D**); SF-EPP after 48 h in PBS at pH 5.5 (**B**,**E**) or in PBS at pH 7.4 (**C**,**F**).

The release of EPP from the SF-EPP film was carried out in PBS buffers at two different pH values, 7.4 and 5.5, to simulate the internal tissue and interstitial fluid of the wound bed and the naturally acidic surface of skin [42], respectively. Figure 5 shows that in buffer solution at pH 7.4, the percentage of released EPP increased progressively up to 8 h until a plateau that did not result in 60% of the total release in 48 h. A similar trend occurred in samples placed at pH 5.5, the only difference being the total amount of released EPP did not exceed 30%. These results are in line with previous studies concerning the development of polymeric films for delivery of antioxidant compounds to the skin [43,44] and demonstrate that the SF-EPP films we prepared are able to release suitable amounts of EPP for acceptable antioxidant activity. The in vitro release data were analyzed with various semi-empirical release kinetics (Korsmeyer–Peppas and Peppas–Sahlin) [45,46]. In order to understand the mechanisms of release from films, R_2_ values were used to identify the best-fit model. The semi empirical models revealed the types of diffusion. In particular, the Korsmeyer–Peppas model is useful for understanding the release mechanism. Indeed, according to the n value (in the case of thin films), the release mechanism has different modes of diffusion as follows: (i) n < 0.5 for Fickian diffusion, (ii) 0.5 < n < 1.0 for anomalous diffusion (combination of diffusion and matrix swelling) and (iii) n ≥ 1.0 for non-Fickian diffusion. On the other hand, the Peppas–Sahlin model allows the determination of the k1 and k2, corresponding to the Fickian kinetic constant and to the matrix swelling kinetic constant, respectively. If the ratio of |k1|/|k2| is higher than 1, drug release is mainly determined by diffusion; if it is lower than 1, the release is mainly determined by matrix swelling. As reported in Table 3, the Korsmeyer–Peppas and Peppas–Sahlin models showed interpolation with R_2_ values greater than 0.90 for both samples. In both release conditions, the samples showed n values lower than 0.5 and negative values for the matrix swelling kinetic constant (k2), indicating that the release is controlled by a Fickian-type diffusion. Furthermore, both the kinetic constants, KKP and k1, of SF-EPP at pH 7.4 are higher than those at pH 5.5, suggesting faster release of the extract from the SF films under physiological conditions. The slower and lesser release at pH 5.5 can be explained with the morphological changes of the matrix at the two different pHs. In this respect, we used SEM analysis to investigate the morphologies of the SF-EPP films after 48 h of incubation in buffer solutions. Figure 6 shows that immersion in the two different buffers for 48 h causes a slight decrease in the thickness of the films (from 51 µm for the pristine SF-EPP film to around 42 and 44 µm after treatment at pH 5.5 and 7.4, respectively), but most importantly, it was possible to observe the formation of aggregates in the treated films. In particular, more aggregates were observed in films incubated at pH 5.5 (Figure 6B). As also reported in the literature [39], this could be linked to the greater presence of β-sheet crystal structures induced by the weakly acidic environment, which being more compact and insoluble in water, could incorporate the EPP, preventing its complete diffusion, giving rise to the slower and lesser release at pH 5.5. This result was validated by ATR analysis (Figure S5) carried out on the SF-EPP films after 48 h of incubation. Indeed, Figure S4 shows a decrease in the intensity of the 1650 cm^−1^ band for the SF-EPP1.5 film incubated at pH 5.5, indicating reductions in the amorphous and water-soluble α-helices and random coil structures.

### 2.7. Cell Viability and ROS Inhibition

**Figure 7:**
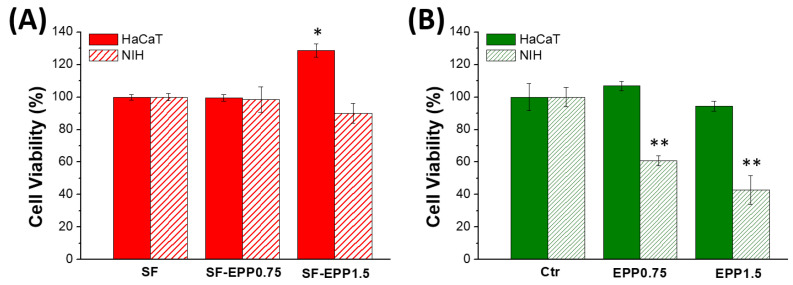
MTT test carried out after 48 h of incubation on HaCaT and NIH cells plated on SF and SF-EPP films (**A**) and on cells treated with free EPP used at the same concentrations present on the films (**B**). Data represent mean ± standard deviation. Statistical analyses were performed using ANOVA followed by Tukey’s test. * *p* < 0.05, ** *p* < 0.01, denote significant differences with respect to the control.

**Figure 8:**
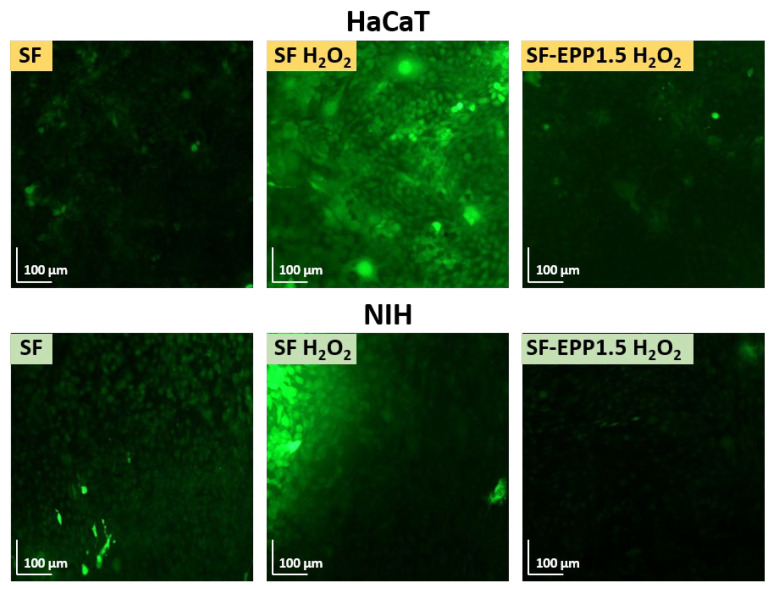
ROS production of HaCaT and NIH cells plated on pristine SF and SF-EPP films before and after treatment with H_2_O_2_. Magnification: 10×, scale bar: 100 µm.

**Figure 9:**
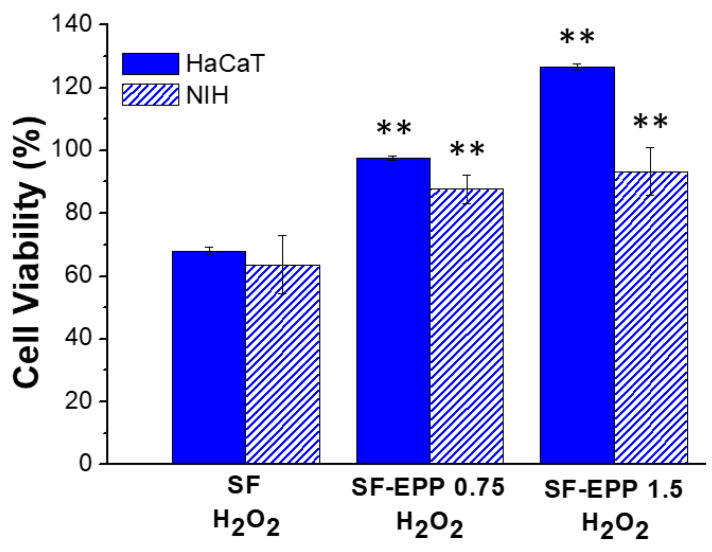
MTT test carried out after 48 h of incubation on HaCaT and NIH cells plated on SF and SF-EPP films and treated with H_2_O_2_. Data represent mean ± standard deviation. Statistical analyses were performed using ANOVA followed by Tukey’s test. ** *p* < 0.01, denote significant differences with respect to the control.

Skin repair is a complex process that involves various cell types that interact with matrix components to re-establish the 3D structure and the functions of the damaged tissue. Before evaluating the radical scavenging activity of fibroblasts (NIH) and keratinocytes (HaCaT) on SF-EPP films, we carried out cell viability assays in order to validate the biocompatibility of our bioactive films with these cell lines. Figure 7A shows that both fibroblasts and keratinocytes, after 2 days of incubation, maintained the same metabolic activity on the SF and SF-EPP films as the control. The resazurin reduction assay carried out at 48 and 72 h (Figure S6) confirmed the maintenance of metabolic activity. Indeed, for each sample, almost 100% cell viability was found for both NIH and HaCaT cells. Noteworthily, for the viability tests, where cells were treated with free EPP at the same concentrations as were loaded on the films, and we observed for NIH cells a reduction in viability that was not found for HaCaT (Figure 7B and Figure S7). Specifically, after 48 h we measured reductions of 40% and 60% with 0.75 and 1.5% EPP, respectively. By using SF-EPP films loaded with the same concentrations of antioxidants, any cytotoxic effect observed in NIH fibroblasts was very likely thanks to the slow release induced by the SF protein. Interestingly, this result demonstrates the feasibility of using our films as matrices for controlled EPP release. Based on these findings, while ignoring any cytotoxic effects of our films, we investigated the antioxidant potential of SF-EPP, evaluating its ability to reduce the intracellular release of ROS after oxidative stress induced by H_2_O_2_. First, we tested the antioxidant activity of free EPP on both cell lines via fluorescence assay. Figure S8 shows that after treatment with H_2_O_2_, ROS production dramatically increased, decreasing only after adding free EPP. As regards cells plated on SF-EPP films, the ROS production was evaluated only qualitatively due to the film’s slight autofluorescence, which did not allow a quantitative analysis. As shown in Figure 8, the intracellular ROS production (directly proportional to the increase in fluorescence of cells; see Section 3) after exposure to H_2_O_2_ was high for both cell lines plated on pristine SF films. Differently, it decreased for SF-EPP1.5, suggesting that EPP released from the SF matrix significantly inhibited ROS production in treated HaCaT and NIH cells, thereby confirming its high antioxidant performance in response to oxidative stress. Finally, the metabolic activity of both cell lines plated on SF and SF-EPP films after exposure to H_2_O_2_ for 48 h was evaluated. As shown in Figure 9, the viability of cells plated on pristine SF significantly decreased after treatment with H_2_O_2_, reaching about 60%. On the other hand, the viability of cells plated on bioactive SF-EPP films was normal (100%).

## 3. Materials and Methods

### 3.1. Pomegranate Peel Powder Preparation

Pomegranate organic fruits (WonderFul variety) were kindly provided by the Horticultural Association (A.P.O.130, Foggia, Italy), and pomegranate peel powder was prepared by the University of Foggia (Department of Agricultural Sciences, Food and Environment), according to literature data [31].

### 3.2. Extraction of Antioxidants from Pomegranate Peel

First, 500 mg of pomegranate peel powder (Figure S9A) was weighed and dispersed in 50 mL of Milli-Q water. Then, the dispersion was sonicated at room temperature for 60 min, filtered and finally centrifuged at 4000 rpm for 10 min to separate the supernatant from the bottom body. The obtained EPP yellow solution (Figure S9B) was frozen and lyophilized using the MODULYO instrument (Edwards), resulting in about 350 mg of EPP powder (Figure S9C).

### 3.3. HPLC-DAD-MS Analyses of EPP

HPLC-DAD-MS analyses were performed on a HPLC Dyonex Ultimate 3000 equipped with a diode array UV detector and mass spectrometer TSQ Quantum Access Max with an electronspray ionization source. Samples 0.5 mL in size were used as sources for the automated injection. LC-MS grade acetonitrile was purchased from Sigma-Aldrich in the highest available purity and was used without any further purification. Ultrapure water (resistivity 18.2 MΩ/cm at 25 °C) was produced in our laboratory by means of a Millipore Milli-Q system. The chromatographic separation was performed on a reverse phase Zorbax Eclipse XDB-C8 column 4.6 × 150 mm, 5 µm, at flow rate of 0.5 mL/min; linear gradient HCOOH 1% aqueous solution/ACN from 95:5 to 20:80. Mass data acquisition was performed in negative ionization (ESI-) and full-scan mode in the range of *m*/*z* 240–1200.

### 3.4. Antioxidant Activity of EPP

The radical scavenging activity of EPP was evaluated by the 2,2-diphenyl-1-picrylhydrazyl (DPPH) assay according to a procedure in the literature [47]. Briefly, 180 µL of EPP in water at different concentrations was mixed with 4 mL of DPPH solution (0.1 mM) in 95% ethanol. The amount of EPP was varied from 10 to 180 µg in each solution. The absorbance decrease at 516 nm was monitored to evaluate the scavenging activity of EPP after storing the samples for 20 min in the dark. The DPPH radical scavenging activity was calculated using the following equation:

(1) $$I\;\left(\%\right)=[1-\frac{A_{\mathrm{sample}}\;-\;A_{\mathrm{blank}}}{A_{\mathrm{control}}}]*100$$

where I is the antioxidant activity (%), A_control_ is the absorbance of the DPPH solution, A_sample_ is the absorbance of the EPP sample mixed with the DPPH solution and A_blank_ is the absorbance of each EPP sample without the DPPH solution.

### 3.5. Preparation of SF-EPP Films

SF-EPP films were prepared by mixing the SF aqueous solution (5% wt/vol) with glycerol (20% wt/wt vs. SF) and EPP (0.75 and 1.5% wt/wt vs. SF). Specifically, EPP powder was first solubilized in water, added to glycerol and then mixed with the SF solution to obtain the weight percentages before mentioned. The obtained blends were dropped on a support of polydimethylsiloxane (PDMS) (1 mL on 1 cm × 3 cm), left to dry under a hood at room temperature and finally piled off of the substrate. The thicknesses of the films were measured by micrometer.

### 3.6. Optical, Structural and Morphological Characterization of SF-EPP Films

A Perkin Elmer Lambda 650 UV–Vis spectrophotometer was used to collect the absorption spectrum of EPP and obtain the percentage transmittance from the free-standing films. Infrared spectra were acquired using the attenuated total reflectance technique (ATR) with a Bruker Vertex 70 interferometer equipped with a diamond crystal single reflection Platinum ATR accessory, in the 4000–600 cm^−1^ region, with 128 scans and a resolution of 4 cm^−1^. The curve fitting of overlapping bands of the infrared spectra covering the amide I and II regions (1450–1750 cm^−1^) was performed using the Levenberg–Marquardt algorithm implemented in OPUS 2.0. Quantitative analysis of the amide I and amide II bands was performed using curve fitting, second derivative and Fourier self-deconvolution methods, as already reported in literature. [48] Spectra were processed using Bruker OPUS software. The amount of α-helices in a sample was computed as the ratio between the sum of the areas of the corresponding peaks in the amide I and amide II bands and the sum of the areas of all of the peaks in the same spectral region.

The thermal properties of the films were measured in a Differential Scanning Calorimetry (DSC) Instrument (METTLER TOLEDO) under a dry nitrogen gas flow of 70 mL min^−1^. The samples were heated at 2 °C min^−1^ from 35 °C to 350 °C. SEM analysis was performed with a Zeiss EVO LS 10 LaB6 scanning electron microscope. For the analysis of films sections, the samples were frozen in liquid nitrogen and then broken in order to induce fragile fracturing. The fractured cross-section was sputtered with a gold layer for 1 min (thickness of gold layer ≈10 nm) and then observed at an acceleration voltage of 5 kV and a working distance of 5 mm.

### 3.7. SF and SF-EPP PBS Stability and Biodegradability

Each film, previously dehydrated at 50 °C for 1 h, was incubated at 37 °C in a 5 mL solution of 1 mg mL^−1^ trypsin (TPCK treated from bovine pancreas, Sigma) in phosphate buffered saline (PBS) at pH 7.4. Each solution contained an approximately equivalent mass (60 ± 2 mg) of films. Solutions were replenished with enzyme and collected daily. At designated time points (1, 3, 5, 7 and 12 days), groups of samples were rinsed in distilled water and prepared for mass balance. Samples were dehydrated in an oven at 50 °C for 2 h. Following removal from the oven, the samples were weighed and returned to new solution with fresh enzyme. Percentage of weight loss over time was determined. Each experiment was performed in triplicate. The pristine SF film was taken as the reference.

### 3.8. Mechanical Characterization of SF-EPP Films

The mechanical properties of the films were evaluated by tensile tests using a Zwick Roell Z1.0 testing machine, with a 200 N static load cell, as previously described [37]. Young’s modulus, tensile strength at break and elongation at break were measured on rectangle-shaped film stripes, obtained by a cutting machine, the length and width of which were 100 and 5 mm, respectively. Before testing, a first sample batch was equilibrated for 72 h at 33% relative humidity (RH) in a container using magnesium chloride saturated solution at 23 ± 2 °C; a second sample batch was equilibrated for 24 h at 80% RH in the climate chamber at 37 ± 2 °C. All specimens were mounted in the film-extending grips, and the tests were carried out at a rate speed of 100 mm min^–1^ until breaking. The RH and temperature of the testing environment were held at 23 ± 5% and 20 ± 2 °C for the first batch and 37 °C and 80% RH for the second batch using the climate chamber. At least five replicate samples were analyzed. The data were elaborated by the TestXpert V11.0 Master software (Zwick Roell, Ulm, Germany). 

### 3.9. Drug Release

Antioxidant release from SF-EPP1.5 film was investigated by dipping samples in PBS buffers at pH 5.5 and 7.4. The film was kept shaking at 37 °C, and aliquots of 1 mL of PBS buffer were withdrawn at appropriate time intervals, and an equal amount of fresh buffer was added (sink conditions). The amount of antioxidant released in the medium from the loaded SF-EPP films was evaluated via UV–Vis absorption band at 372 nm, using appropriated calibration curves. EPP-free SF films were used as controls. The experiments were performed in triplicate. The release mechanism was studied through the curve-fitting analysis of the EPP release profiles, using different mathematical models and carried out by ORIGIN 8.1^®^ software (OriginLab Corporation, Northampton, MA, USA).

### 3.10. Cell Culture

Mouse embryonic fibroblast (NIH-3T3/GFP) and human skin keratinocyte (HaCaT) cells were cultured under standard conditions in DMEM medium, supplemented with 10% (*v*/*v*) FBS, 2 mM L-glutamine, 0.1 mM MEM non-essential amino acids (NEAA), 100 U mL^−1^ penicillin and 100 U mL^−1^ streptomycin, in a humidified incubator set to 37 °C with 5% CO_2_. Cells were seeded on keratin in 24-well plates at a density of 105 cells per cm^2^.

### 3.11. Cell Viability via Resazurin Reduction Assay and MTT Assay

Resazurin reduction assay: The reagent is an oxidized form of the redox indicator that is blue in color and non-fluorescent. When incubated with viable cells, the reagent is reduced, and it changes its color from blue to red, becoming fluorescent. Briefly, cells were seeded on extract pomegranate peels (EPP), SF films and SF-EPP films with complete medium. After incubation, the resazurin reagent was added directly to culture medium—10% by volume of medium was contained in each sample—and incubated for 3 h at 37 °C with 5% CO_2_. Subsequently, aliquots from each sample were transferred to a 96 multiwell plate for fluorescence measurements at λ_exc_ 560 nm and λ_em_ 590 nm (Thermo Scientific Varioskan Flash Multimode Reader). We included a negative control of only medium without cells to determine the background signal and a positive control of 100% reduced resazurin reagent without cells. 

MTT assay: Measuring the intracellular reduction of tetrazolium salts into purple formazan by viable cells [49]. Cells were incubated with MTT solution (5 mg mL^−1^) for 2 h at 37 °C, 5% CO_2_. Subsequently, the MTT solution was discarded, and dimethyl sulfoxide (DMSO) was added to each well. Optical density (OD) was read on a microplate reader at 550 nm (Thermo Scientific Varioskan Flash Multimode Reader). Cell viability for each treatment was calculated as the ratio of the mean OD of replicated wells relative to that of the control. All data were provided as mean ± standard deviation. 

### 3.12. Cellular Treatment

In order to estimate the antioxidant potential of EPP films for the skin, the cells were pretreated (for 48 h) with different concentrations of EPP and SF-EPP films before exposure (for 1 h) to 200 µM of H_2_O_2_. At the end of incubation, cell viability of the samples was analyzed with the MTT assay, as previously described [50].

### 3.13. Measurement of Reactive Oxygen Species

ROS levels were evaluated by means of the probe 2′,7′-dichlorofluorescin-diacetate (H2DCF-DA) [51]. The cells were seeded on extract pomegranate peels (EPP), SF films and SF-EPP films with complete medium for 48 h. At the end of incubation, the cells were treated (30 min) with H2DCF-DA (10 μM) in the dark. Samples were examined using a Nikon Eclipse 80i microscope equipped for fluorescence analysis [52].

## 4. Conclusions

Free standing bioactive films were obtained by mixing a natural protein, such as SF, with antioxidant compounds extracted from pomegranate waste. SF and EPP were both successfully extracted by using eco-friendly methods based on water. UV-Vis and HPLC analyses confirmed the presence of punicalagin and tannins in EPP, and the DPPH test proved its strong antioxidant power. The obtained SF-EPP films were transparent in the visible region, stable in water, had a reduced biodegradability and were flexible at 37 °C and 80% RH. Moreover, an SF-EPP film containing 1.5% wt/wt of EPP, compared to SF, displayed a controlled EPP release based via Fickian diffusion mechanism. Furthermore, in vitro cell viability tests, carried out on fibroblast and keratinocytes, the main skin cells, demonstrated good biocompatibility of the reported bioactive films. Noteworthily, for fibroblast cells, the SF-EPP film showed greater biocompatibility than free EPP, highlighting the importance of having a matrix capable of controlling the release of the active ingredient. Finally, the ROS production test demonstrated the capacity of films to reduce the oxidative stress induced in cells by a common oxidizing agent, H_2_O_2_. All the results collected in this work showed the potential of the SF-EPP film, as a biocompatible, eco-sustainable and bioactive platform, for the treatment of oxidative stress in skin disorders while conforming to the perspective of a circular economy based on waste valorization.

## Figures and Tables

**Table 1:** Mechanical parameters determined at T = 23 °C and 33% RH.

Sample	E (MPa)	TS (MPa)	ε (%)
**SF**	1361.33 ± 39.61	46.91 ± 2.59	6.64 ± 0.20
**SF-EPP 0.75**	1393.54 ± 14.83	43.54 ± 0.65	5.95 ± 0.14
**SF-EPP 1.5**	1428.28 ± 59.90	52.17 ± 4.47	5.88 ± 0.16

The values are expressed as mean ± standard deviation.

**Table 2:** Mechanical parameters determined at T = 37 °C and 80% RH.

Sample	E (MPa)	TS (MPa)	ε (%)
**SF**	119.2 ± 6.5	14.37 ± 7.01	211.1 ± 12.8
**SF-EPP 0.75**	157.8 ± 6.5	19.27 ± 0.24	276.9 ± 0.6
**SF-EPP 1.5**	200.6 ± 8.5	14.34 ± 0.54	229.2 ± 7.3

The values are expressed as mean ± standard deviation.

**Table 3:** Parameters and correlation coefficients for Korsmeyer–Peppas and Peppas–Sahlin models.

Sample	Korsmeyer–Peppas *Q_t_* = *K*_KPt_^n^			Peppas–Sahlin *Q_t_* = *k*_1_*_t_*^0.5^ + *k*_2_*_t_*^1.0^			
	*K*_KP_	n	R^2^	*k*_1_	*k*_2_	R^2^	|*k*_1_|/|*k*_2_|
**SF-EPP pH 7.4**	20 ± 2	0.13 ± 0.01	0.944	18 ± 2	−1.56 ± 0.28	0.973	12
**SF-EPP pH 5.5**	10 ± 1	0.15 ± 0.02	0.904	8 ± 1	−0.48 ± 0.11	0.973	16

## Data Availability

Not applicable.

## Supplementary Materials

The following are available online at https://www.mdpi.com/article/10.3390/ijms23126805/s1.
