# Supplementary material for: Eco-Sustainable Silk Fibroin/Pomegranate Peel Extract Film as an Innovative Green Material for Skin Repair
Source: Int J Mol Sci. 2022 Jun 18;23(12):6805. doi: 10.3390/ijms23126805 (PMC9224408; doi:10.3390/ijms23126805)
Supplement: Supplementary file 1 [file ijms-23-06805-s001.zip › ijms-1723797-supplementary.pdf]

Supplementary Information

Eco sustainable Silk Fibroin/Pomegranate Peel Extract Film as Innovative Green Material for Skin Repair

Marianna Barbalinardo,[a,1] *Marta Giannelli,*[b,1] *Ludovica Forcini,*[b] *Barbara Luppi,*[c] *Anna Donnadio,*[d] *Maria Luisa Navacchia,*[b] *Giampiero Ruani,*[a] *Giovanna Sotgiu,*[b] *Annalisa Aluigi,*[b,e] *Roberto Zamboni,* [b,*] *Tamara Posati* [b,*]

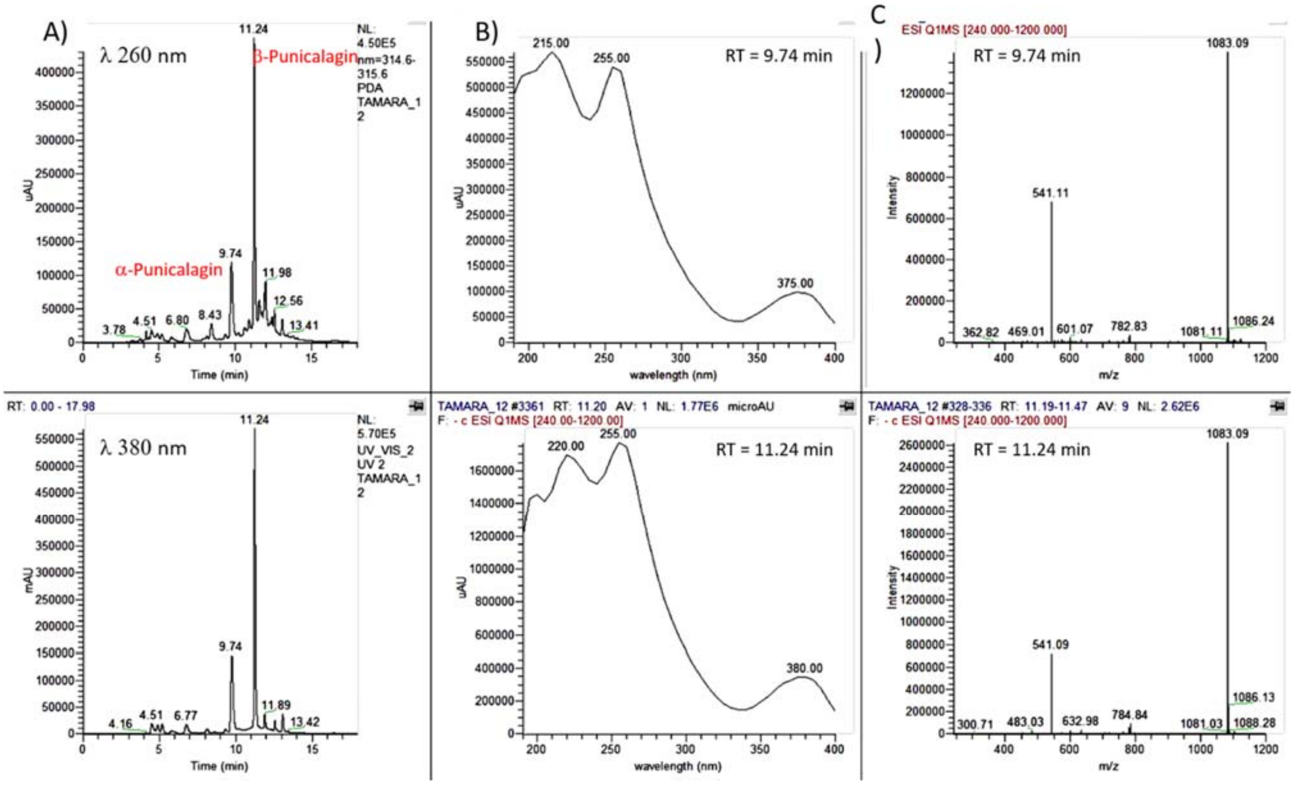

Figure S1. A) HPLC chromatogram of EPP at λ 260 (up) and 380 nm (down); B) UV-vis spectrum of peak at RT = 9.74 min (up) and UV-vis spectrum of peak at RT = 11.24 min (down); C) mass spectrum of peak at RT = 9.74 min (up) and mass spectrum of peak at RT = 11.24 min (down).

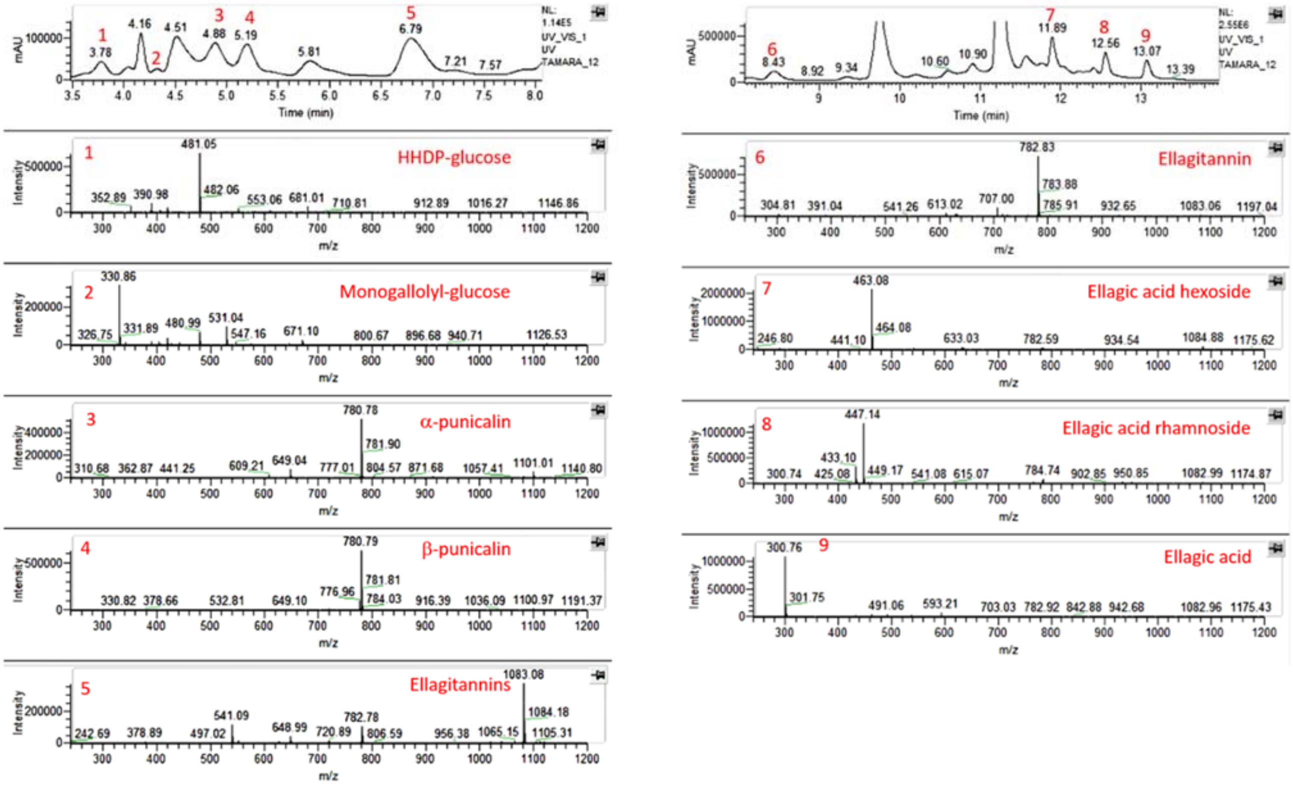

Figure S2. HPLC chromatogram at λ260 nm and MS spectrum of the minor compounds identified in EPP.

Table S1. Retention times RT (min), λmax UV–Vis absorbance, MS (ES-) of tannins identified in EPP.

		RT (min)	λmax UV–Vis (nm)	MS (ES-) m/z
1	HHDP-glucose	3.78	slope	481
2	Monogallolyl-glucose	4.32	275	331
3	α-Punicalin	4.88	781	260-365
4	β-Punicalin	5.19	781	260-380
5	Ellagitannins m/z 1083	6.79	1083	255-375
6	Ellagitannin m/z 783	8.43	783	slope
7	Ellagic acid hexoside	11.89	463	250-360
8	Ellagic acid rhamnoside	12.56	447	250-360
9	Ellagic acid	13.07	301	250-365

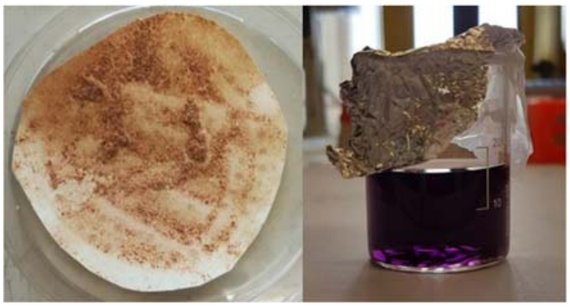

Figure S3. Pictures of extraction residue (left) and DPPH test with ~150 mg of residue (right).

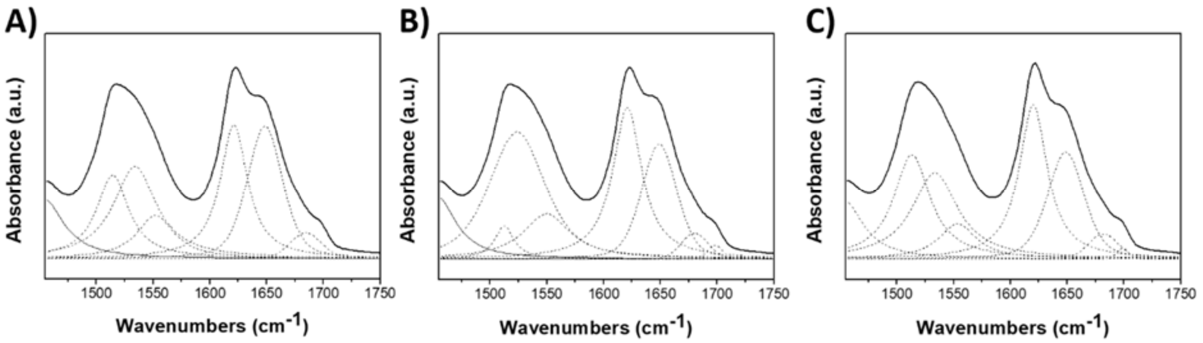

Figure S4. Deconvolution of ATR spectra (Amide I and Amide II bands) of SF (A), SF-EPP0.75 (B) and SF-EPP 1.5 (C).

Table S2. Quantification of the secondary structure elements.

**Centroid (cm^{-1})**	**Area (SF)**	**Area (SF-EPP0.75)**	**Area (SF-EPP1.5)**	**Assignment**
1515	11.10	20.07	15.50	β-structures
1534	16.13	2.56	10.03	α-helix
1550	6.60	8.70	4.33	α-helix
1623	15.03	17.90	19.71	β-structures
1650	15.01	12.52	9.01	α-helix
1700	2.18	2.47	2.64	β-structures

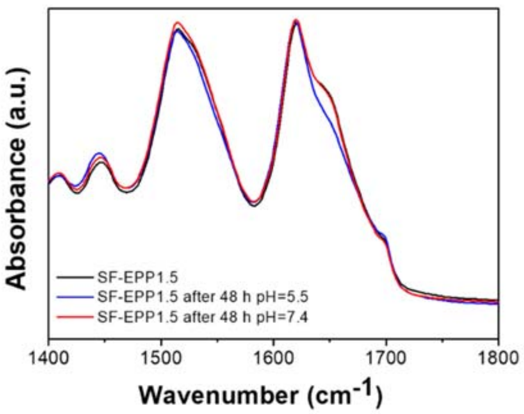

Figure S5. ATR spectrum of pristine SF-EPP1.5 film (black line), SF-EPP1.5 film after 48 h a pH 5.5 (blue line) and SF-EPP1.5 film after 48 h a pH 7.4 (red line) recorded in the 1200−1800 cm^{-1} region.

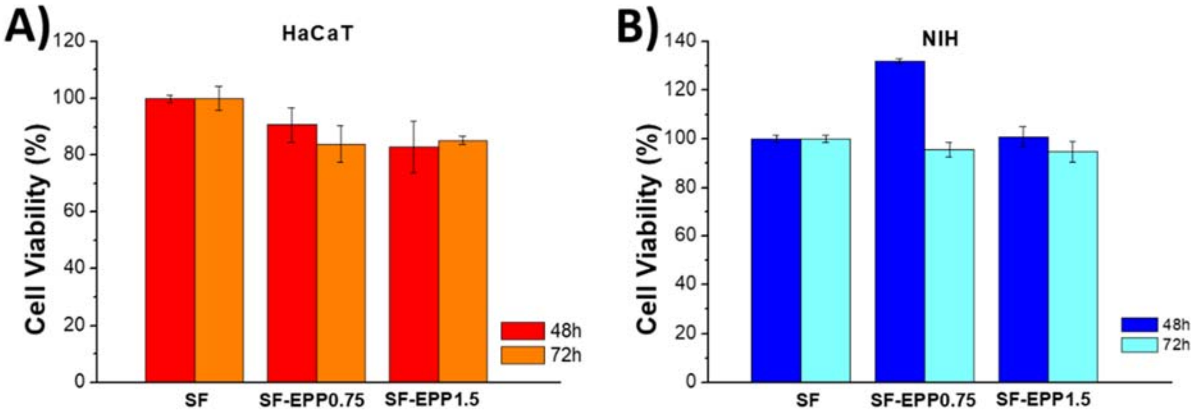

Figure S6. Resazurin assay carried out on HaCaT (A) and NIH (B) cells plated on SF and SF-EPP films.

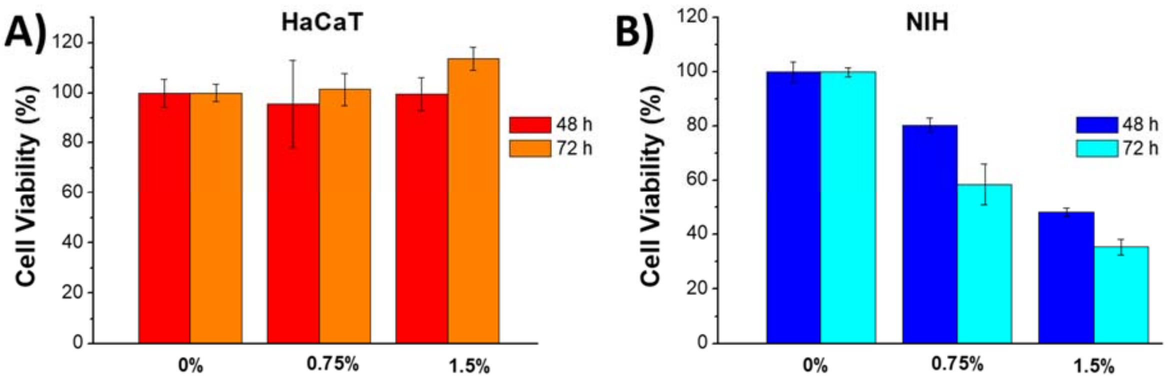

Figure S7. Resazurin assay carried out on HaCaT (A) and NIH (B) cells treated with free EPP.

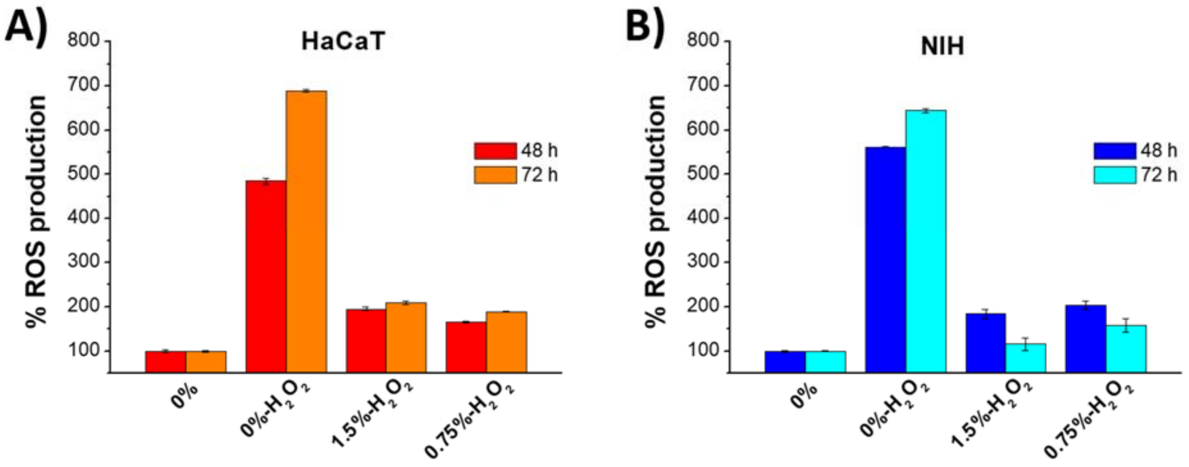

Figure S8. ROS production of HaCaT and NIH cells treated with H_2O_2 with and without adding free EPP.

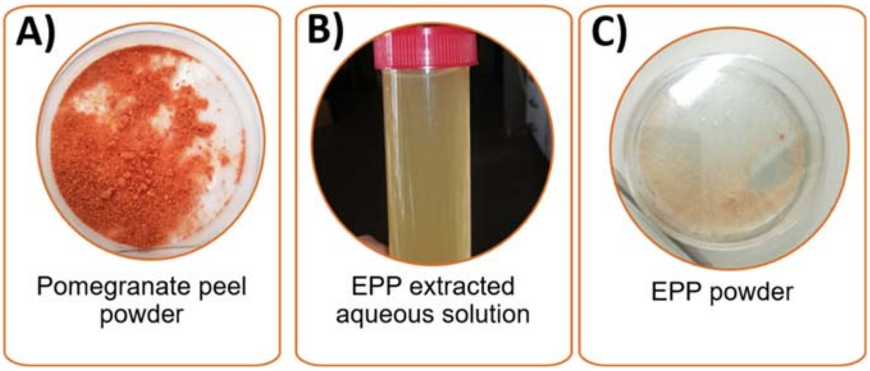

Figure S9. Pictures of pomegranate peel powder (A), EPP extracted aqueous solution (B) and EPP powder (C).
